# Personalisation at the Core of Success: Process Evaluation of the LISTEN Randomised Controlled Trial Evaluating a Personalised Self‐Management Support Intervention for People Living With Long Covid

**DOI:** 10.1111/hex.70270

**Published:** 2025-05-05

**Authors:** Fiona Leggat, Anna Torrens‐Burton, Bernadette Sewell, Nick Sevdalis, Monica Busse, Anne Domeney, Judith Parsons, Maria Ines de Sousa de Abreu, Fiona Jones

**Affiliations:** ^1^ Population Health Research Institute, School of Health and Medical Sciences City St George's, University of London London UK; ^2^ PRIME Centre Wales, Division of Population Medicine, School of Medicine Cardiff University Cardiff UK; ^3^ Swansea Centre for Health Economics Swansea University Swansea UK; ^4^ Centre for Behavioural and Implementation Science Interventions, Yong Loo Lin School of Medicine National University of Singapore Singapore Singapore; ^5^ Centre for Trials Research, School of Medicine Cardiff University Cardiff UK; ^6^ LISTEN Patient and Public Involvement and Engagement Group City St George's, University of London London UK; ^7^ Bridges Self‐Management London UK; ^8^ East Sussex Healthcare NHS Trust Crisis Response Service, St. Annes House St Leonards‐on‐Sea UK; ^9^ Kingston University London UK

**Keywords:** fidelity, implementation, intervention, Long Covid, personalised, process evaluation, self‐management

## Abstract

**Background:**

The development and evaluation of rehabilitation interventions designed to support people with Long Covid (LC) remains an important ongoing priority. Many people with LC experience episodic, debilitating symptoms that can reduce their ability to engage in all areas of activity. The Long CovId personalised Self‐managemenT support co‐design and EvaluatioN (LISTEN) trial co‐designed and evaluated a personalised self‐management support intervention to build confidence and support people to live better with LC. This paper describes the context, implementation, mechanisms of impact and impacts from the LISTEN intervention, in comparison with usual LC services accessed within the National Health Service (NHS).

**Methods:**

A mixed methods process evaluation was nested within the LISTEN pragmatic, multi‐site, randomised controlled trial. Data were collected from sites in England and Wales between September 2022 and January 2024. Observations and focus groups with healthcare practitioners (HCPs) delivering the intervention were conducted to assess fidelity. Standardised implementation measures, focussed on intervention feasibility, acceptability and appropriateness, were gathered from HCPs and intervention participants. Semi‐structured interviews were undertaken with a subset of participants across both trial arms. Data were analysed independently using descriptive statistics, or reflexive thematic analyses, and subsequently integrated, drawing upon the Consolidated Framework for Implementation Research v2.

**Findings:**

Thirty‐six HCPs participated in the process evaluation, and 197 intervention participants completed standardised implementation measures. Across both trial arms, 49 participants took part in semi‐structured interviews. Six integrated themes were constructed from all data sources describing and illustrating links between the context, implementation, mechanisms of impact and impacts: ‘Delivery during uncertainty and ambiguity’, ‘Diversity and consistency of usual care’, ‘Drivers for self‐care and the impact of self‐generated expertise’, ‘Appropriate if unexpected support’, ‘Personalisation at the core of success’ and ‘A spectrum of change’.

**Conclusion:**

The LISTEN intervention is an appropriate, feasible intervention for participants and HCPs. The intervention can be delivered to a high level of fidelity following training and with ongoing HCP support. Access, receipt and perceptions of NHS LC services were variable. Personalised, relational interventions, such as LISTEN, can foster favourable impacts on confidence, knowledge and activity and are acceptable and strongly recommended within LC rehabilitation services.

**Patient or Public Contribution:**

The study was supported by a patient and public involvement and engagement (PPIE) group from project conception to study end. Using their lived expertise, seven people with LC supported accessible recruitment (e.g., materials), data collection (e.g., topic guides), data interpretation (e.g., theme construction and reviewing findings) and dissemination activities (e.g., online webinars).

**Trial Registration:**

ISRCTN36407216, registered 27/01/2022.

## 1 Introduction

### 1.1 Background

Long Covid (LC) is a condition collectively termed and accepted by people experiencing long‐lasting symptoms following Covid‐19 [1, 2, 3]. The most recent UK estimates suggest approximately 1.9 million people may be experiencing LC (also known as post‐Covid condition), with over 700,000 having had symptoms for longer than 2 years [4]. Across all body systems, over 200 LC symptoms have been identified, with fatigue, breathlessness, heart palpitations, cognitive dysfunction and muscle and joint pain prevalent across symptom clusters [5, 6]. The burden of LC is substantial, with 79% of people reporting adverse impacts on daily activities and ability to work [4]. The economic impact related to work absence is great [4, 7], and those living with the condition face not only complex, fluctuating multi‐system symptoms, but also ongoing social and psychological impacts [8, 9, 10, 11]. With ‘invisible’ symptoms, people can experience guilt, stigma, isolation and helplessness [10, 11, 12, 13, 14]. Referred to by some as an ‘end to normality’, people with LC describe being unable to fulfil personal responsibilities, engage in joyful activity and feel like themselves [11].

Across 2020 and 2021, National Health Service (NHS) LC services were set up across the United Kingdom (UK) [15]. Guidance for services includes personalised, safe rehabilitation and self‐management support [16, 17]. However, reports have continued to suggest that the provision, access and availability of LC services are variable [18], with some clinicians unsure how to evaluate and manage people with LC [3], and some services in England and Wales being decommissioned [19].

The Long CovId personalised Self‐managemenT support co‐design and EvaluatioN (LISTEN) trial was funded by the UK National Institute for Health and Care Research (NIHR) in July 2021. The project co‐designed and evaluated a personalised self‐management support intervention for people with LC [20, 21]. Within the co‐design process, narratives and lived experiences were placed centrally, addressing the needs and priorities of people with the condition [11, 20, 22]. A pragmatic, multi‐centre, two‐arm, parallel group, individually randomised controlled trial with sites across England and Wales was subsequently used to evaluate the effectiveness and cost‐effectiveness of the co‐designed LISTEN for people with LC on routine activities, and various secondary outcomes including emotional well‐being, fatigue and self‐efficacy in comparison to usual NHS care [21, 23]. Trial findings are reported elsewhere [23], but, in summary, the LISTEN intervention was found to be effective in enhancing participation in routine activities (*p* = 0.052), self‐efficacy (*p* < 0.001), emotional well‐being (*p* = 0.001) and reducing fatigue (*p* < 0.001) in comparison to usual NHS care. The health economics analysis further found the LISTEN intervention to be cost‐effective from a societal perspective, with participants losing fewer hours of work and requiring less informal care than those receiving usual care [24]. A mixed‐methods process evaluation was nested within the trial. This focussed on understanding the context, implementation, mechanisms of impact and participant and HCP‐reported outcomes and impacts from the LISTEN intervention, in comparison with usual LC services accessed within the NHS. The findings from this process evaluation are reported here.

### 1.2 The LISTEN Intervention

The development of the LISTEN intervention is described elsewhere in accordance with the TIDieR framework [20, 22]. Underpinned by social cognitive theory and self‐efficacy principles [25, 26, 27] and adapted from Bridges Self‐Management (Bridges) [28], the LISTEN intervention drew upon existing evidence and was integrated with real‐life experiences to enhance the knowledge, skills and confidence of people to manage day‐to‐day activities with LC. As the proposed mechanism of impact, key sources of self‐efficacy in the intervention included goal mastery and vicarious peer modelling experiences.

**Figure 1:**
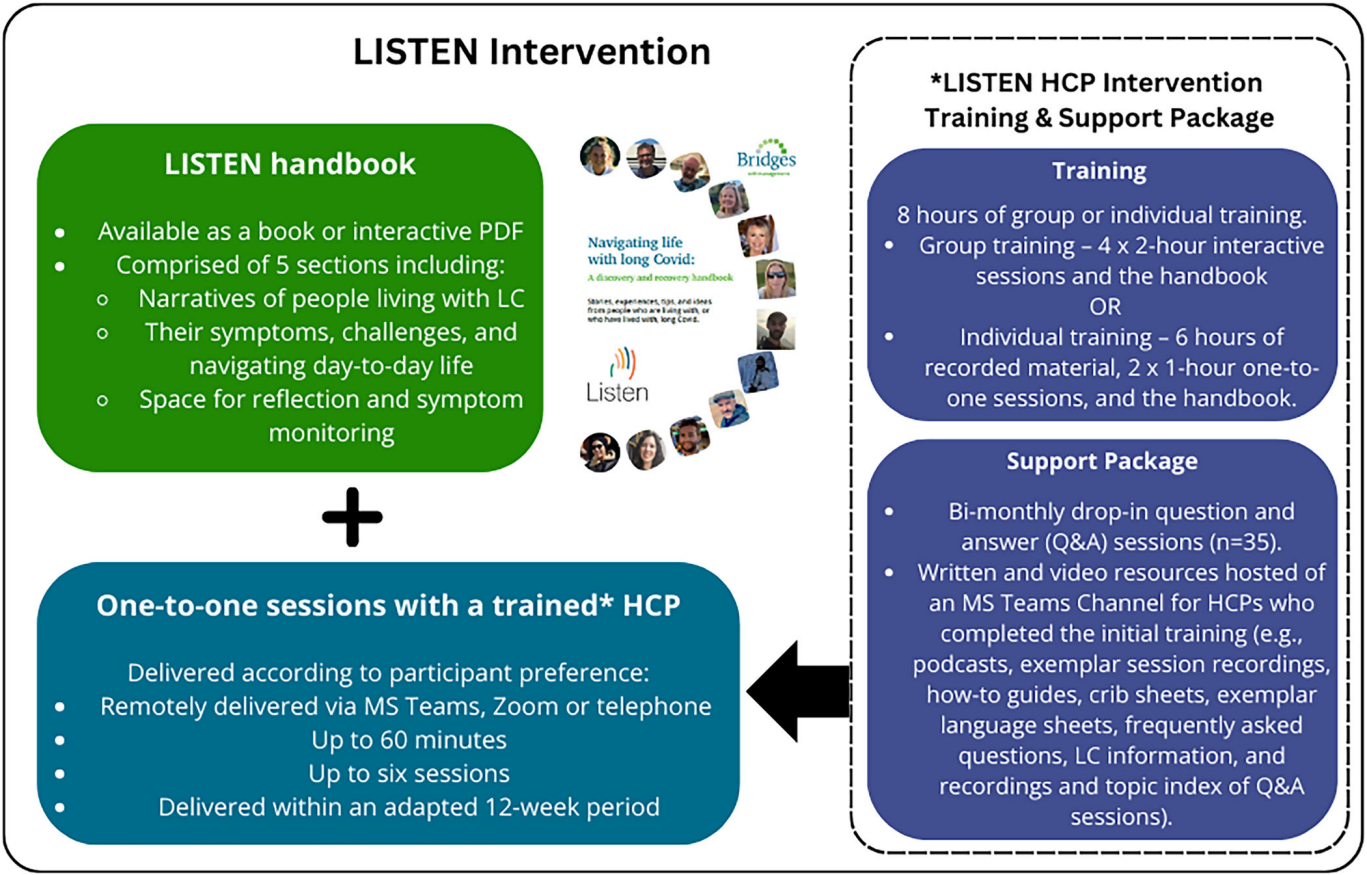
The LISTEN intervention.

Eight core principles underpinned the LISTEN intervention. Developed through co‐design [11, 20, 22], these principles informed two intervention components: the LISTEN handbook and up to 6 one‐to‐one personalised self‐management support sessions with a trained LISTEN HCP (see Figure 1) [22, 23]. Minimum adherence was defined as four sessions [23].

Congruent with Bridges [28], HCPs were trained so their interactions were less prescriptive and more collaborative, supporting individuals' confidence, skills and knowledge to manage daily life with LC. Overall, 72 HCPs (nurses, physiotherapists and occupational therapists) undertook training in the LISTEN intervention principles. To supplement training and maximise intervention delivery fidelity, a ‘wrap‐around’ support package was provided for HCPs [22]. This package provided HCPs with a virtual platform of electronic resources and drop‐in support options (see Figure 1).

### 1.3 Study Purpose

The LISTEN intervention was a complex intervention formed of multiple, interacting components which required skills and expertise to deliver [29]. To evaluate complex interventions, mixed methods process evaluations have been recommended to explore and understand how an intervention is implemented, and why it works, for whom and in what context [29, 30]. This paper reports on the findings of the mixed methods process evaluation embedded within the LISTEN trial. We describe the context (NHS usual care and intervention), the implementation (of the LISTEN intervention and factors influencing this [e.g., fidelity]), the mechanism/s of impact (by which the LISTEN intervention may produce change) and intended/unintended participant and HCP reported outcomes and impacts to illustrate and elaborate on trial findings.

## 2 Methods

### 2.1 Study Design and Theoretical Underpinning

**Figure 2:**
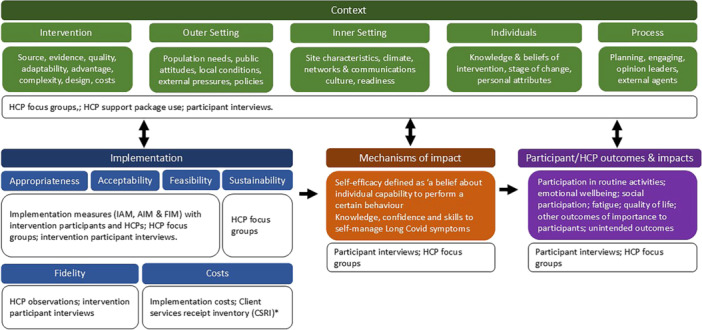
Overview of the theory‐driven LISTEN process evaluation. *Costs are reported elsewhere [24].

Nested within the effectiveness and cost‐effectiveness trial [21, 23], the mixed methods process evaluation was grounded on MRC guidance [29, 30] and the Consolidated Framework for Implementation Research version 2 (CFIRv2) [31, 32] (see Figure 2). The CFIRv2 is a well‐established framework used to understand factors that impact upon successful implementation. Using the CFIRv2, five main determinants of LISTEN implementation were proposed a priori, (i) the LISTEN intervention itself, including its theoretical underpinnings, (ii) the implementation process, (iii) the people involved in designing and implementing LISTEN, (iv) the local context/s (including exploring NHS LC services) in which LISTEN was delivered and (v) the wider context of the NHS and the ongoing pandemic. The Proctor implementation outcome taxonomy, formed of eight constructs (acceptability, adoption, appropriateness, feasibility, fidelity, cost, penetration and sustainability) [33], further informed data collection method selection and implementation outcomes explored (see Figure 2). A convergent design with a qualitative weighting (QUAL + Quan) was used to situate, explain and explore the trial findings in more detail [34, 35]. The research was underpinned by ontological relativism and epistemological constructivism. The full protocol, including information on the usual care comparator group, is published elsewhere [21].

### 2.2 Participants

Trial participants were aged 18+, had lived with LC symptoms for over 12 weeks and had not been hospitalised with Covid‐19. Participants were able to self‐refer to the study, while others were invited by HCPs during routine care. A subset of participants from both arms of the trial (LISTEN intervention and usual care) were recruited to the process evaluation. Maximum variation criterion‐based sampling was used to recruit a diverse group reflecting characteristics of the UK LC population. Core criteria included gender, age, ethnicity and spread across sites/geographical locations to explore usual NHS care. All HCPs involved in LISTEN intervention delivery at participating sites across England and Wales were invited to participate [21, 23].

### 2.3 Data Collection and Sampling

Multiple data collection methods were used to support the theory‐driven process evaluation (see Figure 2) [21]. A summary is given below.

#### 2.3.1 HCP Observations

Intervention fidelity was assessed through observations of 10% of recorded one‐to‐one remote LISTEN intervention sessions. Recorded via online platform (e.g., MS Teams) or telephone, HCPs' language and behaviours were reviewed against a fidelity checklist containing the eight core intervention principles (plus reference to/use of the LISTEN handbook). The checklist was used for collective analysis across a maximum of six sessions delivered by an HCP for one participant (*n* = 25 HCPs [10% total intervention group recruitment target/six sessions = 25]). Each of the core skills in the fidelity checklist were rated by the observer using a 3‐point Likert scale from 1 to 3 (1 = not observed, 2 = partially/inconsistently observed and 3 = consistently observed). Purposive maximum variation criterion‐based sampling was used for HCPs based upon site, number of intervention sessions delivered and HCP background. Observations were undertaken during trial delivery, and 20% were observed by an additional member of the research team.

#### 2.3.2 Implementation Measures

To assess appropriateness, acceptability and feasibility of the LISTEN intervention, all intervention trial participants (*n* = 277) were invited to complete implementation scales following intervention receipt at 3 months post‐randomisation [21]. All HCPs delivering the intervention were invited to complete the same scales at 3‐ and 6‐months post their intervention delivery start date [21]. The scales, developed from Proctor's taxonomy [33], were the Acceptability of Intervention Measure (AIM), the Intervention Appropriateness Measure (IAM) and Feasibility of Intervention Measure (FIM) (4 items each; 12 implementation items in total; all scored on 5‐point Likert scales) [36]. The scales have acceptable content validity and structural validity [36].

#### 2.3.3 HCP Support Package Use

All HCP attendances at bi‐monthly Q&A sessions were recorded to explore the use of the available LISTEN wrap‐around support during intervention delivery. Attendance frequencies were calculated and used for analysis.

#### 2.3.4 Semi‐Structured Interviews

Remote, semi‐structured interviews were conducted with trial participants following completion of all outcome measures (3 months post‐randomisation [21]). Interviews with the LISTEN intervention group explored participants' experiences and any outcomes and/or impacts from the intervention. Interviews with usual care participants explored similar topics but focussed upon the content and experiences of NHS LC services accessed and/or any alternative support (see Supplementary Information file 1).

#### 2.3.5 Focus Groups

Remote focus groups were held with HCPs who delivered the LISTEN intervention. Topics included their experiences with intervention training and the support package, intervention delivery and contextual factors (e.g., health service setting) (see Supplementary Information file 2). HCP sampling replicated that used in the observation data collection.

### 2.4 Data Analysis

Process evaluation data were analysed independently of and before the trial statistical analysis. Preliminary analyses were undertaken with each data source independently before integration [37].

#### 2.4.1 Preliminary Analyses

All interview and focus group data were transcribed verbatim and uploaded to separate NVivo files to enable the initial coding of raw data. Thematic analyses were used to develop themes from shared patterns of meaning within the datasets [38, 39]. Following familiarisation through re‐reading transcripts, inductive codes were applied to the data. Inductive codes facilitated data‐driven, interpretative coding within a loose deductive framework informed by MRC process evaluation guidance and the CFIR v2 (see Figure 4). Following independent researcher coding, researchers debated codes, and through collaboration with the research team, shaped rich, theoretically informed, data‐driven themes. Themes were subsequently refined and named.

HCP observations, HCP support package use and the AIM, IAM and FIM were analysed using descriptive statistics. To calculate HCP fidelity scores from observations, ratings from the eight core intervention principles were summed for each HCP (maximum = 24). HCPs support package use was calculated from frequency counts of actual Q&A attendance from possible Q&A sessions (e.g., total possible within the period of trial involvement). Both measures were converted into percentage scores.

The AIM, IAM and FIM scores were summed individually to calculate a score for each scale (minimum = 4, maximum = 20). Higher scores on each scale indicate a more overall implementable intervention.

#### 2.4.2 Integration and Synthesis

**Figure 3:**
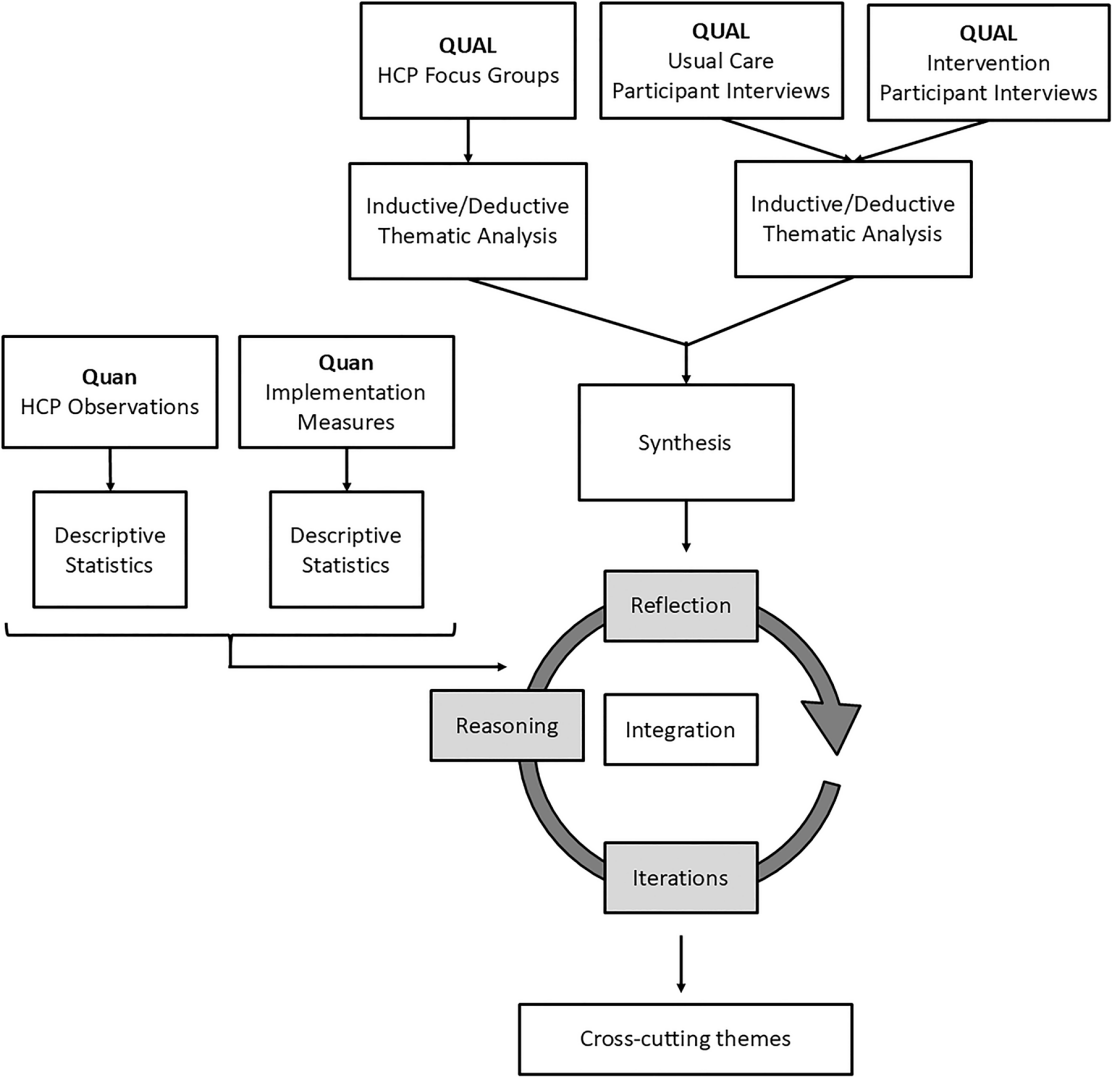
Overview of the data source analyses and integration.

**Figure 4:**
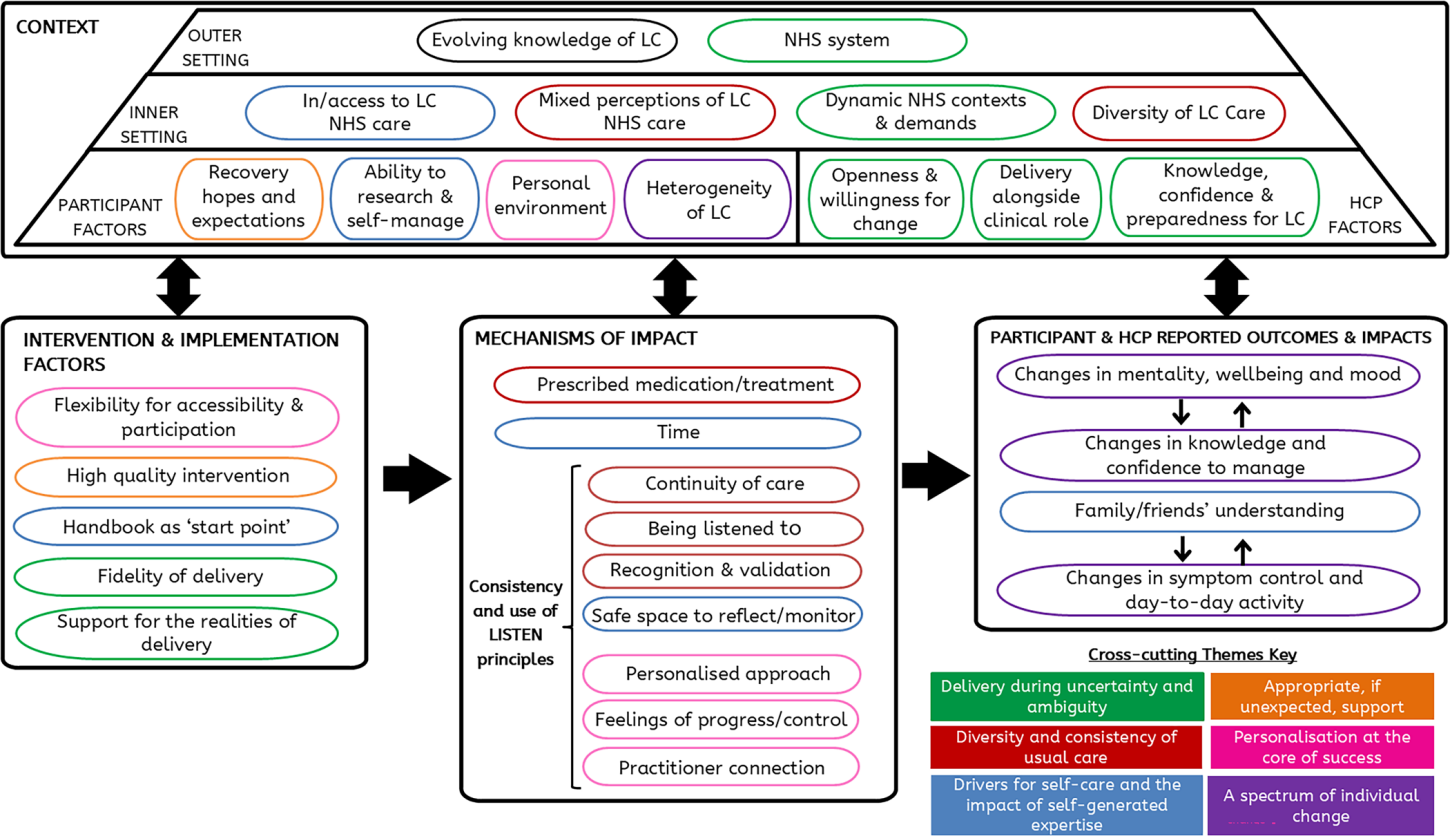
Overview of cross‐cutting theme construction from the analysis and integration process. *‘Context’, ‘Intervention & implementation factors’, ‘Mechanisms of impact’ and ‘Participant & HCP reported outcomes & impacts’ boxes (black outlines) comprise the coding framework informed by MRC guidance and the CFIR. **Smaller boxes inside each black‐outlined box represent integrated themes from all data sources (interviews, focus groups, observations, implementation measures, support package use). ***Cross‐cutting themes, reflected by a coloured outline per theme (green, orange, red, pink, purple, blue; see figure key), constructed from the relationships between integrated themes.

Qualitative and quantitative data were integrated to explore nuances and possible explanations for the trial findings within and between datasets, to increase the value and usability of the findings [37] (see Figure 3). Qualitative and quantitative data were integrated using an inductive/deductive hybrid thematic analysis (QUAL + Quan) [35]. Firstly, existing themes from the independent focus group and interview analyses were organised using the MRC guidance and the CFIR v2 (see Figure 2). Following this, the content of each theme was explored, and collectively, integrated themes were developed through revision, adaptation and synthesis to illustrate shared patterns of meaning and prevent any duplication from the two sources. Through a process of iteration, reasoning and reflection, quantitative data was integrated into the themes. Following further reflection and re‐visiting of the raw data, cross‐cutting themes were constructed from the theoretically informed integrated themes to show variance, interplay and complexity and illustrate relationships pertinent to the LISTEN logic model [21] (see Figure 4). Initial integration was undertaken by the lead author, but multiple members of the research team and PPIE group were involved during the synthesis cycle. Following this, external reflections were sought from the wider LISTEN team.

## 3 Results

Overall, 56 HCPs from across sites delivered the intervention, and 36 of these participated in the process evaluation. Of those, 25 were observed and received a fidelity score, 27 participated in focus groups, and 38 and 26 completed the AIM, FIM and IAM at 3‐month and 6‐month post LISTEN implementation (see Table 1). Focus groups averaged 113 min each in duration.

The AIM, FIM and IAM were completed by 197 intervention participants. The demographics of the 49 participants who took part in the process evaluation interview are shown in Table 2. Interviews averaged 56 min each.

**Figure 5:**
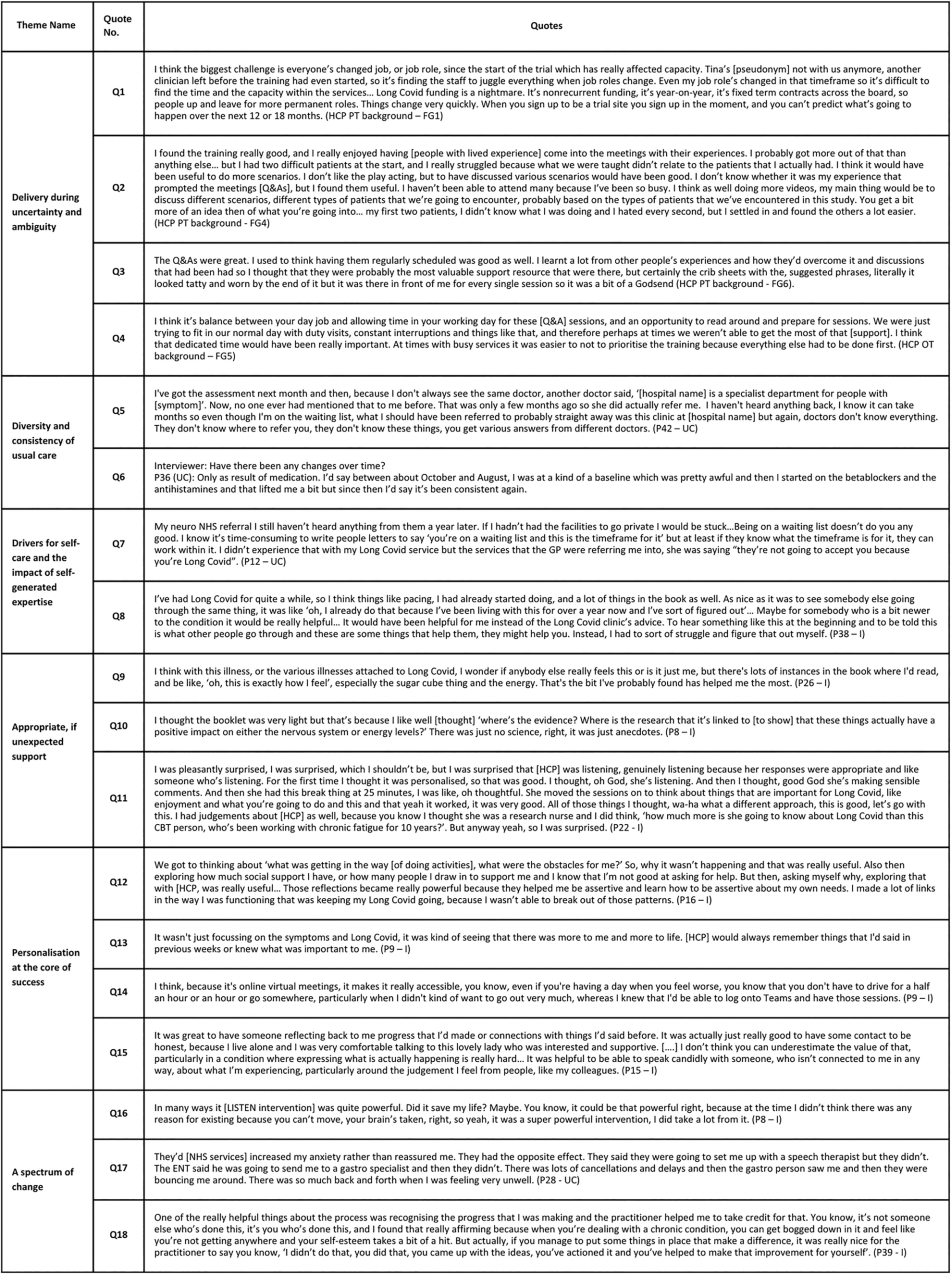
Quotes from interviews and focus groups illustrating the six cross‐cutting themes.

Integrated findings from all qualitative and quantitative data sources, informed by MRC guidance and the CFIRv2 are shown in Figure 4 (see Supplementary Information file 3 for data sources). These findings comprise six cross‐cutting themes which illustrate relationships between contextual factors, participant and HCP factors, intervention and implementation factors, mechanisms of impact and participant and HCP reported impacts and outcomes. Quotes illustrating each theme are presented in Figure 5.

**Table 1:** HCP demographics.

	HCP observations	HCP focus groups	AIM, FIM and IAM 3‐month	AIM, FIM and IAM 6‐month
Gender				
Male	5	6	5	4
Female	20	21	33	22
HCP background				
Physiotherapy (PT)	9	10	10	9
Occupational therapy (OT)	6	4	7	4
Nursing	6	8	13	9
Psychology	3	3	4	2
Other	1	2	4	2
NHS region				
Wales	7	7	12	7
England	18	20	26	19

**Table 2:** Participant demographics.

	Interviews	AIM, FIM and IAM
Sex at birth		
Male	20	54
Female	29	135
Preferred not to disclose	0	8
Trial group		
LISTEN intervention	25	197
Usual care	24	N/A
Age		
≤ 34	4	19
35–44	10	47
45–54	17	57
55–64	7	46
65–74	9	23
≥ 75	1	5
Ethnicity		
White Welsh/English/Scottish/Northern Irish/British	35	168
White Irish	2	4
Any other White background	4	10
Mixed or multiple ethnicity	2	5
Asian ethnicity	4	9
Black/African/Caribbean background	1	0
Other ethnicity	1	1
NHS region		
England	36	123
Wales	13	74

### 3.1 Delivery During Uncertainty and Ambiguity

This theme, derived from HCPs experiences, relates to their delivery of a new, non‐prescriptive, personalised intervention within a demanding healthcare service. With only fixed‐term periods of LC funding, HCPs described how NHS services were unstable, liable to changes and had high staff turnover due to fixed‐term contracts. Working within this evolving environment, dedicating time to prioritise, prepare and deliver LISTEN was challenging for some HCPs (Q1, Figure 5).

Following LISTEN training, all HCPs reported gaining useful knowledge of the eight core intervention principles. The training was perceived to be engaging and appropriate, whether delivered in a group or individual format, and gave HCPs dedicated time to engage, away from any other responsibilities. However, the intervention represented a shift in their way of working from their usual ‘expert’ positioning to a collaborative person‐centred practice. Some resonated with and absorbed the principles, whilst others struggled with not being prescriptive. Some HCPs felt a lack of confidence to deliver, unaware of what to expect and how to apply the principles. This was especially apparent for HCPs with little LC experience, who felt the training did not fully prepare them for the complexities of the condition (Q2, Figure 5).

HCPs' engagement with the ‘wrap‐around’ support varied. Q&A attendance averaged 16% (Md), ranging between 0% and 79%. However, HCPs who delivered sessions to one or two participants (*N* = 25) averaged 8% attendance (Md), compared to 35% (Md) for HCPs who delivered to three or more participants. Overall, HCPs described how the ongoing support enhanced their knowledge, confidence and skills to deliver, through valuable opportunities to gain peer support, share experiences and problem‐solve challenges (Q3, Figure 5). With many competing demands, the need for dedicated time to access support when preparing for intervention delivery was expressed (Q4, Figure 5).

**Figure 6:**
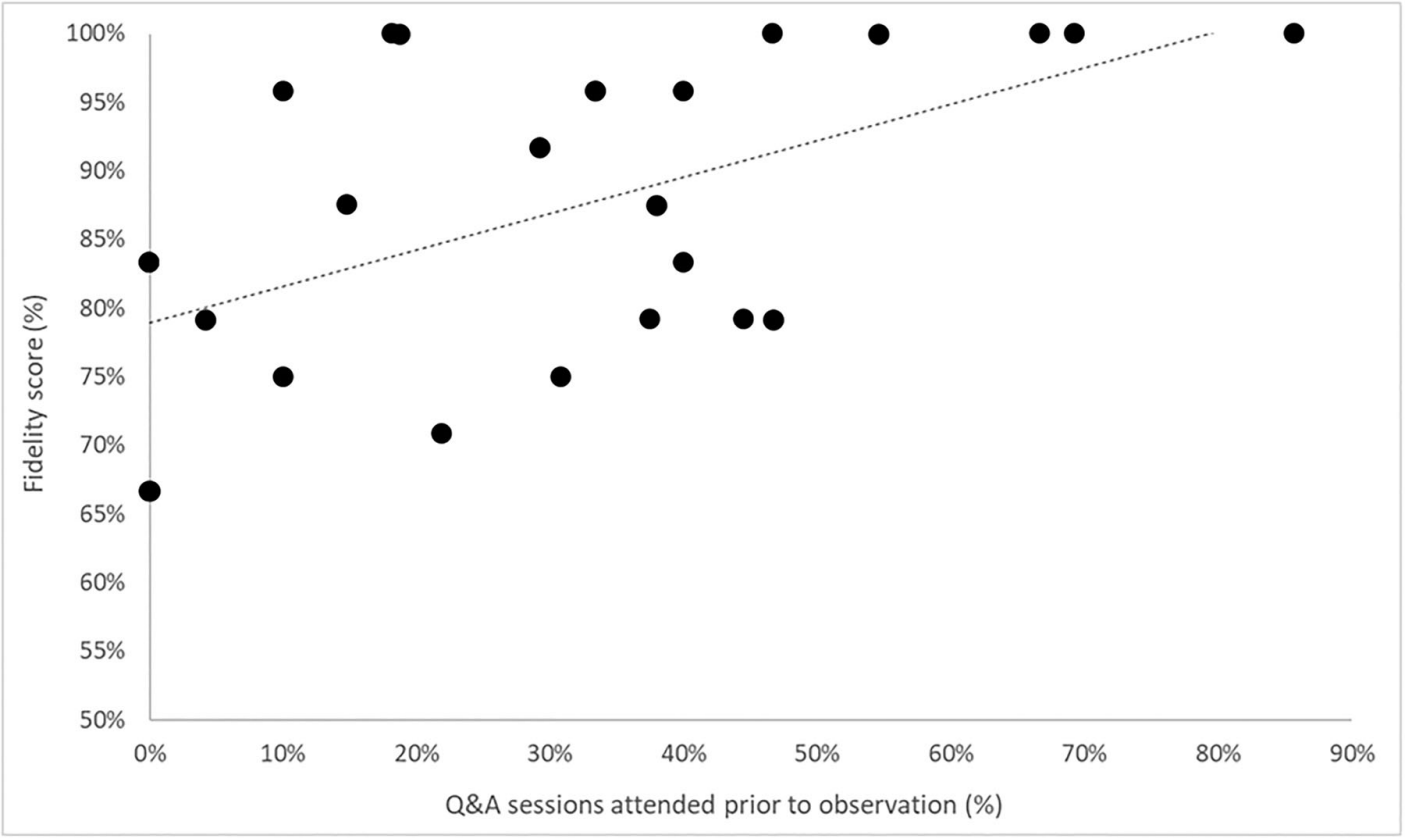
Scatterplot showing association between Q&A session attendance (before observation) and intervention delivery fidelity scores.

Fidelity of the intervention sessions delivered averaged 88% (Md), ranging between 67% and 100%. This showed that, based on sessions observed, most HCPs delivered the intervention as intended. This was echoed within participants' interviews; favourable outcomes reported were often attributed to intervention principles. HCPs with higher fidelity scores were often those who attended a greater number of support sessions (see Figure 6), or had greater engagement with the written resources, but also those with fewer clinical responsibilities. Overall, HCPs regarded the intervention as feasible to deliver (see Table 3), but this may be contingent on the ongoing support provided which supplemented initial training.

**Table 3:** Implementation data median scores (and IQRs). Possible score range 4–20.

		AIM	IAM	FIM
HCPs	3‐month	17 (16–20)	16 (14–19)	17 (15–19)
	6‐month	16.5 (16–19)	16 (16–19)	16 (15–18)
Participants	3‐month	17 (16–20)	16 (16–20)	16 (16–19)

Abbreviations: AIM = Acceptability of Intervention Measure, FIM = Feasibility of Intervention Measure, IAM = Intervention Appropriateness Measure.

### 3.2 Diversity and Consistency of Usual Care

HCPs and participants in both trial arms shared experiences of different LC healthcare services. The diversity of LC services spanned primary and secondary care (e.g., general practitioners [GPs], respiratory, cardiology and LC clinics), different specialisms (e.g., physiotherapy, nutrition and psychology), different formats (e.g., face‐to‐face, virtual, group and one‐to‐one), different assessments/scans and different treatments/advice (e.g., medication, exercise, supplements and education). Despite the variety of services, participants from both groups described challenges obtaining diagnoses, appointments and referrals. Here, some GPs were considered to lack the requisite knowledge needed on LC services and pathways (Q5, Figure 5). For those who managed to access services, sometimes through persistence, family or HCP colleague support, the LC label from GPs was considered facilitative.

Perceptions of services were inconsistent in the usual care group. Multiple examples of unsatisfactory care included experiences of one‐off appointments, unsuitable or generic prescribed activities/medications/advice, services focusing on one symptom, and dismissal from HCPs or multiple referrals for different services. Participants described how these experiences could lead to negative changes in their mood and well‐being and deterioration in mental health, isolation, uncertainty and hopelessness.

Some attributes of NHS care mirrored the core principles of the LISTEN intervention (e.g., listening, validation and continuity of care). In similarity with the LISTEN intervention, care continuity (including provision of any ongoing care), validation, being heard and willingness to find advice from HCPs and NHS services were perceived positively. When experienced, these behaviours enabled participants to feel better about their condition. Some prescriptions and treatments also met participants' needs and led to short‐term favourable changes in symptom severity or prevalence (Q6, Figure 5).

### 3.3 Drivers for Self‐Care and the Impact of Self‐Generated Expertise

This theme concerns participants' drivers to and ability to self‐manage their condition, and the implications of such self‐generated expertise on perceptions of the LISTEN intervention. While some participants from both groups had accessed NHS services and gained benefits, many had struggled. Some cited difficulties with travelling to appointments and a lack of local provision. Some participants were dismissed from their GPs due to a lack of symptom severity or if their symptoms did not match the local services provision. Waiting times and the energy required to chase services further inhibited NHS access (Q7, Figure 5).

Some participants across the groups turned to private care or self‐directed research. Private services sought included physiotherapy, nutrition, acupuncture and hyperbaric oxygen therapy. Many participants searched websites and social media to find strategies and the latest scientific developments. Participants in the usual care arm described trying multiple strategies for symptom relief, and some used logs or diaries to monitor any changes. Those engaging in self‐monitoring, also encouraged within the LISTEN intervention, perceived it to be helpful for recognising symptom changes. Over time, some intervention and usual care participants gained knowledge of how to self‐manage their condition.

Perceptions of the LISTEN handbook were influenced by participants' level of pre‐existing knowledge and ability to self‐manage. Those with existing knowledge and skills considered the handbook more of a ‘start point’, already aware of the ideas and strategies suggested, but a useful resource for family and friends to better understand LC (Q8, Figure 5). Instead, intervention participants who had not met others with LC, nor engaged in social media and who were open to learning from others, found greater value in the handbook stories and information.

### 3.4 Appropriate, if Unexpected Support

For most intervention participants, the handbook was considered high quality, trustworthy and appropriate for their needs. While some considered stories to be overly positive, most identified with people in the book and found the hints and strategies of others useful (Q9, Figure 5). However, framed as ‘support to help live better with LC’, some participants instead desired a medical treatment to relieve symptoms or offer a cure. These mismatched expectations resulted in several participants withdrawing from the intervention, while for others, the book was not considered scientific enough (Q10, Figure 5). Although weblinks to research and reputable sources were provided within the handbook, some links caused frustration when they became outdated and could not be accessed.

Many intervention participants considered the one‐to‐one support sessions with HCPs helpful and the most valuable intervention component. Some were initially unsure, but over time, they felt sessions were high quality (Q11, Figure 5). In a context where science was perceived to be ‘catching up’, HCPs and participants reported the intervention offered acceptable and appropriate support while a cure was found (see Table 3).

### 3.5 Personalisation at the Core of Success

Participants in both usual care and intervention groups considered personalisation and flexibility as two crucial principles of effective care. Intervention group participants consistently described how intervention sessions were personalised to their needs. Participants enjoyed building a relationship and working collaboratively with their HCP to address what mattered most to them. The LISTEN intervention's core principles were often attributed to outcomes reported by HCPs and intervention participants. These included being listened to, and having someone who wanted to get to know them, encourage them to do enjoyable activities, help them to understand their own condition and think about why strategies do or do not work and help them to feel a sense of progress (Q12, Figure 5).

Although considered highly valuable by participants in both groups, personalised care was not a consistent feature of NHS services. Usual care participants described receiving support from HCPs who were unfamiliar with their condition and circumstances, often with a focus on one symptom per appointment. With interrelated symptoms and other life priorities, this was often considered inappropriate. The holistic approach of the LISTEN intervention was considered more beneficial (Q13, Figure 5).

Intervention participants and HCPs also valued the flexibility of the intervention, including the ability to dip in and out of the handbook as preferred. Similarly, the choice of session duration and frequency facilitated participation and minimal intervention session dropout, although work commitment was occasionally a barrier for participants. Short breaks, turning cameras off and the choice of virtual or telephone delivery further facilitated participation and helped when navigating severe symptoms. The accessibility and flexibility options enabled participants to feel safe and comfortable in their own homes, save their physical and cognitive energy, and opt for their preferences (Q14, Figure 5).

Social support from family and/or friends influenced the value placed on the personalisation and flexibility of the intervention and NHS services. Compared to those with minimal support, participants with close ties who provided tangible support and understood their daily challenges had more positive perceptions of NHS services; family could advocate or support with travel. Conversely, intervention participants without social connections found the LISTEN HCP relationship particularly facilitative to their well‐being (Q15, Figure 5).

### 3.6 A Spectrum of Change

This theme relates to the variety of outcomes experienced by participants across both groups. These outcomes relate to three main areas: (a) well‐being and mood, (b) knowledge and confidence to manage and (c) symptom control and ability to undertake day‐to‐day activity. Changes described by participants and witnessed by HCPs were influenced by factors including self‐management expertise, intervention expectations and symptom severity and prevalence.

Positive changes to well‐being and mood were reported by all intervention participants and were often attributed to the relational components of one‐to‐one sessions, and sometimes to the handbook. Changes reported spanned a range of areas, including greater acceptance of their LC, a more positive outlook and hope for the future, and improved mental health (e.g., less anxiousness and less guilt). For some, improvements were short‐term (e.g., mood), and for others, longer‐lasting (e.g., altered outlook). Some improvements were also more profound than others (Q16, Figure 5). Participants who were initially apprehensive of the intervention or perceived the handbook to have a ‘lack of science’ also described positive well‐being changes from participation. The dedicated time to offload, be heard by and reflect with an HCP supported validation, mood and improvements in well‐being, and reduced the emotional burden placed on family and friends. Usual care participants felt similar validation and support from HCPs, but this was inconsistent. When support was delayed or unavailable, mood and well‐being could be negatively impacted (Q17, Figure 5). For many intervention participants, changes started with a greater feeling of well‐being. Through validation and feeling heard, intervention participants described being able to focus on personal learning (e.g., understanding their symptom triggers) and develop strategies to manage their symptoms and capacity to do everyday activities. As knowledge and confidence of their LC improved, so did mood and feelings of well‐being. Through personal learning, intervention participants described a greater confidence in their ability to control symptoms, overcome setbacks, and navigate life with the condition.

Although some outcomes described were not dissimilar to the usual care group, they were attributed differently. Intervention participants described learning and integrating strategies to foster long‐term change, including recognising progress and developing a ‘personal tool kit’ for ongoing management. Such confidence and skills were perceived to be valuable outcomes from the intervention (Q18, Figure 5). In contrast, the usual care group tended to acquire more generic knowledge, obtained didactically from HCPs or found through self‐directed research. Knowledge was often symptom‐specific (e.g., linked to heart rate, breathing control, etc.), as opposed to holistic personal learning. Changes in symptoms in the usual care group were attributed to trial and error of strategies, or from prescribed treatments and medications. For those juggling multiple strategies simultaneously, it was challenging to relate success to a particular strategy.

Across both groups, some participants reported no symptom or activity level change. Some of these individuals tended to have stable or milder symptoms with limited room for change. Others, in the intervention group, felt the limited number of intervention sessions inhibited the opportunity for change and reduced their motivation and accountability to persist with supported self‐management strategies after sessions were completed. Most participants across both groups felt that they would still seek further medical support as needed due to the ongoing scientific advancements in LC.

Overall, intervention participants reported more holistic changes to their everyday lives to live better with LC, whereas the usual care group reported some changes to single symptoms.

## 4 Discussion

This study reports the findings from the nested mixed methods process evaluation of the LISTEN randomised controlled trial. The LISTEN trial found a co‐designed, personalised self‐management support intervention to be more effective in supporting people with LC to live better with the condition when compared with usual NHS LC care [23]. Process evaluation findings provide a unique insight as to why the intervention was effective, for whom and how intervention experiences compared with usual NHS LC care. Themes also highlight key considerations for NHS LC services and future implementation.

### 4.1 Comparison With Previous Literature

Overall, HCPs delivering the intervention found it to be feasible and appropriate after initial training. LISTEN HCPs found the approach different to their usual practice and, consistent with previous findings, sometimes found the uncertainty of the condition challenging [40] (e.g., the inability to give answers or recovery timeframes). Importantly, confidence to deliver was enhanced by access to a comprehensive support package. By offering a platform for support, LISTEN HCPs formed a unique community of practice and generated experiential knowledge which supported intervention delivery fidelity. Despite juggling competing demands, most HCPs found the time and space to deliver intervention sessions in adherence to the core intervention principles. Although training is considered within MRC frameworks when developing and reporting complex interventions [29], findings reinforce the need for dedicated training and support time to maximise intervention delivery fidelity and HCP confidence.

As reported elsewhere [3, 18, 41], usual NHS LC care experienced by this group was extremely variable. LC clinics varied in size, staffing, modality of delivery and service provision, reflecting the skills of HCPs leading the service, although most included medical investigations [18, 42]. Participants' perceptions of services were also mixed. As highlighted previously, investigations, treatments and continuity of care, albeit limited, were perceived favourably, while uncommunicative services, restricted access and long waiting times were considered barriers [40, 41, 42, 43]. The ‘Pathways to Care Model’, developed in the STIMULATE ICP study, describes how the GP has two roles in the route to LC care: recognising and reacting to the problem [43, 44]. For participants in LISTEN, barriers were recognised at both points. Participants reported how some GPs did not recognise or validate their condition, nor did they understand how to support people with managing LC or signpost to LC services. Instead, many participants cited persistence as a strategy to successfully access any NHS service. These findings replicate other studies illustrating that patients can struggle to overcome the first hurdle of getting into a care pathway [11, 40, 43, 44].

This study provides explanations of the trial findings and highlights the impacts of the LISTEN intervention on participants' well‐being, knowledge and confidence and day‐to‐day activity [23]. These changes mirror some found within other LC intervention studies across the UK and Germany [45, 46, 47], but collectively, the LISTEN trial and process evaluation results align with existing evidence that LC support is more effective when personalised to individual experiences and symptoms.

Self‐management programmes underpinned by self‐efficacy principles have been shown to support people with long‐term conditions [48, 49]. The LISTEN findings reinforce this. The LISTEN logic model proposed self‐efficacy, ‘a belief about individual capacity to perform a certain behaviour’ as an anticipated causal mechanism [21]. Aligning with this logic model, mastery (e.g., trying new strategies) and vicarious experience (e.g., seeing others' stories in the handbook) were embedded within the intervention core principles. Increases in self‐efficacy were clearly identified within the clinical trial findings [23] and provide support for elements of this intervention as an effective method of enhancing self‐efficacy, combined with subsequent impacts on symptom control and participation in daily activity. Collectively, the trial and process evaluation findings add to existing evidence for understanding how LC rehabilitation interventions need to be personalised to individual experiences and symptoms.

Findings further indicate that changes in self‐efficacy could be self‐perpetuating. Improvements in knowledge and confidence to navigate life with the condition led to greater feelings of symptom control and increases in day‐to‐day activity. However, such changes in symptom control and activity also appeared to enhance well‐being for intervention participants. This may be attributed to increased opportunities to engage in joyful activity restoring feelings of identity [11]. Combined, LISTEN findings highlight that self‐efficacy can be a powerful mechanism of change in supporting people to live better with LC [23].

Fang et al. found that space for personal reflection, within an understanding environment, helped people to make sense of, justify and adapt to the disruption in their personal narratives, caused by LC [50]. Our findings were similar. In both trial arms, being heard, gaining a connection with an HCP and provision of a safe space for discussion, free from stigma and judgement, were considered beneficial for well‐being and further positive outcomes. Even when changes in knowledge, confidence and/or symptom control were not described, improvements in well‐being and mood were attributed to key intervention components (e.g., attentive listening and being curious) by all intervention participants. This strongly supports how relational and personalised mechanisms (e.g., connection and validation) can explain changes to well‐being and mood in the absence of any activity improvements.

### 4.2 Implications

Findings from LISTEN offer several points of key learning for NHS LC services and rehabilitation programmes.

Firstly, there is a need for personalised, holistic care. Intervention participants valued receiving care specific to their needs and building a relationship with an HCP who wanted to get to know them as a person, not just their symptoms. Provision of generic or siloed support from services limited people's ability to understand and self‐manage their own unique condition, yet, through quality attentive listening, HCPs personalised support and facilitated problem‐solving, reflection, personal learning and strategy development. This study also revealed how people can have wide variations in LC knowledge and that such knowledge can impact intervention perceptions. Therefore, to support the population of people with LC, interventions and support packages need to be flexible and adaptable. Personalised approaches offer such flexibility and thus suit people who have lived with the condition for many years, as well as people recently diagnosed. Dose and format of care should also be personalised where possible. Providing options, including remote delivery, can support people in overcoming symptom‐related challenges [45, 46], and in LISTEN, this flexibility contributed to favourable feedback from participants who were able to control their engagement. Person‐centred care is a marker of quality in LC services [18], which findings from this study strongly support. Therefore, post‐Covid services across England and Wales should seek to maximise the delivery of personalised care where possible to support people in living better with LC.

Secondly, self‐management that is not only personalised, but also supported, is warranted within NHS care pathways. Self‐management is still considered by some to be signposting, information‐giving and education which has limited effectiveness [51, 52]. Instead, our findings show the need for HCPs to provide personalised and supported self‐management, a concept gaining focus within the management of other complex conditions such as stroke rehabilitation [53, 54]. Through the provision of time to reflect and problem‐solve with an HCP, many intervention participants experienced improvements in personal learning, problem‐solving skills and control of their condition. Rather than providing short‐term relief for specific symptoms, the intervention supported people to construct their own unique, personal strategies for navigating their LC. This has important implications for NHS services when considering the sustainability of impacts from, and cost implications of, supported self‐management versus ‘traditional’ self‐management. Recommendations advocate for multidisciplinary, integrated care and rehabilitation [3, 17], but owing to the complexity, and psychosocial impacts of physical LC symptoms, medical symptom‐specific treatments alone may not be sufficiently impactful.

Finally, to optimise the impact of providing personalised, supported self‐management, HCPs must feel confident and knowledgeable to deliver language and strategies rooted in the core intervention principles. Reported in other studies, HCP LC training was limited at the onset of the condition [40, 41]. This contributed to HCP's lack of clinical knowledge and confidence, especially if symptoms fell outside of their usual remit [42]. Although NHS training programmes have since been developed [55], our findings place an emphasis on training which is co‐delivered by people with lived experience to illustrate real‐life examples, the complexities of the condition and the language preferences. To deliver and embed the language and skilled strategies necessary to deliver supported self‐management, HCPs will also require more than just a one‐off training package. Alongside exemplar materials and support resources, HCPs will need organisational flexibility to structure and deliver supported self‐management sessions personalised to patient needs. LC is complex and episodic, and post‐Covid services may need to be flexible with their own service targets and outcomes, to enable the needs of people with LC to be centred and fully addressed.

Findings from LISTEN provide the first evidence to suggest that an LC intervention combining existing theory and evidence, together with the collective experiences of people, can enhance the activity, self‐efficacy and well‐being of people with the long‐term health condition. The LISTEN intervention will now be refined into an implementation package that has the potential to spread and scale across NHS services. Existing training and support programmes for HCPs should highlight the complexity of the condition and offer opportunities to develop key language and strategies in line with the LISTEN core principles. Through further participant engagement, updates to the LISTEN handbook will be undertaken to include a greater variety of stories, symptoms and solutions to meet the differing knowledge and day‐to‐day needs of the LC community. The transferability and applicability of the LISTEN intervention will also be explored to understand if and how core principles may support people living with other complex long‐term conditions.

## 5 Strengths and Limitations

The LISTEN process evaluation utilised multiple methods to provide a rich, comprehensive exploration of the context, implementation, mechanism/s of impact and intended/unintended outcomes. This included interviews with the usual care group participants to explore provision and access to NHS LC services (experiences of the comparator). Data sources were synthesised to create theoretically informed cross‐cutting themes to illustrate relationships and nuances within findings.

Since April 2024, NHS post‐Covid services have been delivered by Integrated Care boards providing support for people with long‐term conditions [16]. This process evaluation was completed before this change in delivery, and thus, it was not able to explore the implementation of LISTEN in this different context. With changing services and the growing burden placed on the NHS, further research is needed to understand how the LISTEN intervention can be implemented into organisations and studied at scale to sustain a personalised, supported approach to LC self‐management.

Further work to explore how the intervention may work, why and in what context for people from non‐white, non‐English/Welsh speaking backgrounds is also needed. Only eight interview participants were from non‐white ethnic backgrounds. Therefore, while these intervention participants found LISTEN appropriate and described favourable outcomes, these findings may not be transferable or representative of other diverse and marginalised groups. Intervention adaptations may be necessary for the intervention to be feasible and acceptable for different communities.

## 6 Conclusion

Access to self‐management support within LC rehabilitation programmes has been recommended by NICE [17]. Yet, LISTEN is the first trial to explore the effectiveness of a personalised self‐management support intervention for people with LC across England and Wales. The LISTEN intervention was appropriate and acceptable to participants and HCPs. The personalisation and relational components of the intervention were key mechanisms of impact, leading to favourable outcomes, with greater self‐efficacy recognised as both a mechanism and an outcome. Usual care variation led to mixed outcomes for those in the control arm of the study. Whilst the findings do not represent a cure, they demonstrate how a personalised self‐management intervention can offer more suitable and effective support to some of the 1.9 million people in the UK living with LC in comparison to some existing NHS care.

## Author Contributions

**Fiona Leggat:** writing – original draft, investigation, methodology, writing – review and editing, formal analysis, conceptualisation, data curation, project administration. **Anna Torrens‐Burton:** methodology, formal analysis, investigation, data curation, validation, writing – original draft. **Bernadette Sewell:** conceptualisation, funding acquisition, writing – review and editing, methodology. **Nick Sevdalis:** conceptualisation, funding acquisition, writing – review and editing, methodology, formal analysis. **Monica Busse:** conceptualisation, funding acquisition, writing – review and editing, methodology, writing – original draft. **Anne Domeney:** writing – review and editing, methodology, formal analysis. **Judith Parsons:** writing – review and editing, methodology, formal analysis. **Maria Ines de Sousa de Abreu:** writing – review and editing, methodology, conceptualisation, funding acquisition. **Fiona Jones:** writing – original draft, conceptualisation, funding acquisition, investigation, methodology, writing – review and editing, formal analysis, validation.

## Ethics Statement

This study was approved on 13 December 2021 by the Research Ethics Committee (REC) for Wales (Wales REC 7), recognised by the United Kingdom Ethics Committee Authority (UKECA), REC reference 21/WA/0368.

## Consent

All trial participants and HCPs provided informed consent to participate in this study. For trial participants, informed consent was gathered through an electronic REDCap database before participation as part of the randomised controlled trial. Participation in the process evaluation was optional for trial participants. For HCPs, informed consent forms were sent via email, signed electronically and returned through email. All participants were provided with the right to withdraw from the study at any point without giving a reason. All consent forms are stored securely in a university password‐protected drive accessible only to the research team and will be destroyed after a period of 10 years. All procedures were followed in accordance with the Declaration of Helsinki.

## Conflicts of Interest

F.J. is the founder and CEO of Bridges Self‐Management, a non‐profit social enterprise that was involved in the co‐design of the LISTEN intervention and training of LISTEN healthcare practitioners. M.B. was recently appointed (25 April 2024) as a non‐executive board member of Bridges Self‐Management. M.B. was a member of the NIHR Health Technology Assessment Commissioned Funding Committee from 2020 to 2023 and is a current member of the NIHR Advanced Fellowship panel and MRC Clinical Fellowship Panel. N.S. is Chief Editor, Frontiers in Health Services, Implementation Science Section.

## Supporting information

Supplementary_File_1.

Supplementary_File_2.

Supplementary_File_3.

## Data Availability

The data that support the findings of this study are available from the corresponding author upon reasonable request. Data requests undergo a review process to ensure that the request complies with patient confidentiality, regulatory and ethical approvals and any terms and conditions associated with the data.
