# Supplementary material for: Personalisation at the Core of Success: Process Evaluation of the LISTEN Randomised Controlled Trial Evaluating a Personalised Self‐Management Support Intervention for People Living With Long Covid
Source: Health Expect. 2025 May 5;28(3):e70270. doi: 10.1111/hex.70270 (PMC12050411; doi:10.1111/hex.70270)
Supplement: Supplementary file 1 — Supplementary_File_1. [file HEX-28-e70270-s001.pdf]

Listen

LISTEN Process Evaluation: Interview Guide for People living with Long Covid

Section 1: Introductory questions

Please could you share your experiences of living with long Covid?

- How would you describe your journey with long covid up until this point?
- What challenges have you faced?

What motivated you to get involved with this research?

Section 2: Intervention/usual care receipt

Please could you describe what care you have received?

Usual care experienced - control

- What was provided?
    - Frequency
    - Duration (of sessions)
    - Who provided this?
    - Content of the app/sessions/materials?
- Where did you receive the care?
- How was the care initiated?
    - Was it always on your request or were you followed up?

Intervention receipt/fidelity – (Intervention group)

Please can you describe your understanding of the intervention?

- What do you know about the resources?

Please could you describe how the intervention was introduced to you by the practitioners?

- What did they say about the resources?
- Please could you talk me through your first session?

Please could you explain what you did with the practitioner in the sessions?

- How did you feel after each session?
- Where were the sessions held with you?
- When were the sessions held with you?

In the sessions, how do you feel the practitioner…?

- listened to you and your experiences (skill 1 and 2)?
- tried to understand who you are as a person (skill 2 & 3)?
- helped you to feel more in control with managing everyday life (skill 4)?
- helped you to find small steps to move forwards? (skill 5)
- encouraged you to reflect upon what has worked and why? (skill 6)
- explored what means most to you? (skill 7)
- helped you to build joy into your life? (skill 8)
- Please describe how you used the LISTEN book? (intervention) Are there any specific examples you can share?
- What did you think of the book as a resource?

Section 3: Experience of/using the intervention/care - feasibility

What is your perception of the quality of the intervention/care services?

Was participating in the intervention feasible for you? If no, why not? (intervention)

Have you used any other materials/strategies alongside the intervention? If yes, what? (intervention)

Section 4: Barriers/facilitators to use/understanding of the intervention/care

What do you feel facilitated or helped you understand/use the intervention/care services?

- Why?

What do challenges did you face understanding/using the intervention/care services?

- Why?
- Did you overcome these challenges? If yes, how?

Prompts could include:
Individual factors (e.g., did your symptoms/confidence/motivation help/inhibit use? How?)

Intervention (e.g., did the design/trustworthiness/quality of intervention help/inhibit use? How?)

Process (e.g., did the delivery of the intervention help/inhibit use? How?)

Outer setting (e.g., did your changing needs/public perception help/inhibit use? How?)

Section 5: Outcomes of care/intervention – acceptability/appropriateness

How appropriate was the intervention/care for your needs?

- Do you feel as though you need any further medical/health care going forwards?

How do you feel the intervention/care satisfied your needs for managing everyday life living with long covid?

- If yes, in what ways did it help/satisfy you?
- Compared to how you felt before the intervention, how has it impacted your…?
    - Everyday life and/or symptoms?
    - Confidence?
    - Knowledge of and ability to use new strategies?
    - Feeling of control?
    - Ability to, or productivity at work
- What part of the intervention/care was most helpful?
    - Why was this part the most helpful?
- What part of the intervention/care was least helpful?
    - Why was this part the least helpful?

To encourage reflection on outcomes, example questions to prompt participants (drawn from the intervention) could include:
What's one small thing you have learnt about yourself from the intervention?

What's one small thing from this experience that has benefitted you?
How do you feel now?
What will you take forward from this experience?
What have you been able to do now that you were unable to previously?
How confident do you feel about managing your condition in the future?
What changes have you noticed? Why do you think those have happened?
