# Supplementary material for: Personalisation at the Core of Success: Process Evaluation of the LISTEN Randomised Controlled Trial Evaluating a Personalised Self‐Management Support Intervention for People Living With Long Covid
Source: Health Expect. 2025 May 5;28(3):e70270. doi: 10.1111/hex.70270 (PMC12050411; doi:10.1111/hex.70270)
Supplement: Supplementary file 2 — Supplementary_File_2. [file HEX-28-e70270-s002.pdf]

LISTEN Process Evaluation: Focus Group Guide for Rehabilitation Practitioners

Section 1: Introductory questions

Please could you tell me about your role and everyday responsibilities as a rehab practitioner?

- Did you have personal experience supporting people with long covid before the trial? Clinical/friends/family/self.

Section 2: Developing contextual understanding of the site/context

Please tell me about your site and your team

- Size, relationships with colleagues, leaders, communication, perceptions of new ideas & change.

What existing long Covid service do you have at your site?

- What is provided?
  - Frequency? content? duration? by whom? where? how was this initiated?
- How does the intervention compare to this service?
- How has this changed over the course of the trial?

What motivated you and the site to participate in the clinical trial?

- Was joining the trial considered an important priority?
- Were you aware of any other settings implementing the intervention?

Section 3: Implementation process - delivery/fidelity/feasibility

Training

Do you remember the 8 key skills from the intervention training? If yes, what were they?

If you had to describe the training to somebody else, what would you say?

What were your perceptions of the training?

Was the training you received appropriate? Do you feel it met your needs?

- Could it be improved? If yes, how?
- Was there anything missing?
- How did it make you feel about delivering the intervention?

Implementation

How ready would you say your team/service was to introduce LISTEN?

- What was most helpful, or the biggest hindrance?
- If you were to re-introduce LISTEN, what would you do differently? (or if you were asked to advise another site to introduce LISTEN, what would be the single most important piece of advice you would give them).

Please describe how you implemented the intervention.

- Did you have an implementation plan? Did you have an implementation lead or champion or team? If yes, what was their role / what did they do?

Did you/your unit/your organization set goals related to the implementation of the intervention?

- Were these monitored?
- Were these achieved?

What parts of the wrap around support package did you access? (intervention)
- What parts of the package did you find most/least useful?
- Why did you access that? How did it meet your needs?
- How much time did you spend using/how frequently did you access the support package?
- What parts do you think would be essential for practitioners delivering LISTEN in the future?

<u>Delivery</u>

Imagine I am a patient, please can you introduce the intervention to me?

Please could you describe what you did in the first session?

Please can you describe what you did in the following sessions?

How feasible was the intervention delivery?

Were any modifications to the delivery needed? If yes, what were those modifications?

How did you use the book when working with a participant? (intervention)

- What sections did you use?
- What did you find helpful/unhelpful when using the book with participants?

<u>Section 4: Barriers/facilitators to delivery</u>

What do you feel facilitated intervention delivery (including use of the book) with patients?
- Why?

What do you feel prevented or hindered intervention delivery (including use of the book) with patients?
- Why?

<u>Prompts could include:</u>

Inner setting factors (e.g., leadership support, peer support, size of team, time, intervention compatibility with the culture, learning climate)
- Consistency/fit with existing daily work/everyday practices.
- Support from PI, site staff and other practitioners at site.
- Intervention fit with the values and norms of the site.

Personal factors (e.g., motivation/confidence)
- Training – knowledge/confidence/skills
- Motivation

Participant factors (e.g., their needs/understanding).
- Participants symptoms impact on sessions.
- Participants past experiences with long Covid services
- Participants attitudes/beliefs towards intervention
- Participant DNAs/session attendance

Intervention factors (e.g., complexity)
- Quality/design of the intervention
- Source of the intervention – credibility of co-design.

- Ease/complexity of intervention to implement.

Section 6: Perceived Outcomes

Do you feel the intervention was effective in supporting people living with long covid?

- If yes, why do you think this?
  - Did it meet patients' needs?
  - Did it produce the desired benefits? (Enhance confidence, mastery, control for managing long covid)
- How/Why do you think the intervention was effective?
  - What parts of the intervention do you think were most helpful for patients?
  - What parts of the intervention do you think were least helpful for patients?

Do you feel the intervention has impacted you, or your future practice working with long covid patients?

Section 7: Sustainability/Scalability

When the trial concludes, and assuming LISTEN is found effective, what would you do to ensure it continues to be delivered in your service?

If you were asked to implement LISTEN nationally, what would you put in place to ensure it is implemented as intended to achieve effective clinical outcomes?

- What would you recommend/change?
