# Supplementary material for: Personalisation at the Core of Success: Process Evaluation of the LISTEN Randomised Controlled Trial Evaluating a Personalised Self‐Management Support Intervention for People Living With Long Covid
Source: Health Expect. 2025 May 5;28(3):e70270. doi: 10.1111/hex.70270 (PMC12050411; doi:10.1111/hex.70270)
Supplement: Supplementary file 3 — Supplementary_File_3. [file HEX-28-e70270-s003.pdf]

	Usual care interviews	Intervention interviews	HCP Focus Groups	HCP Observations	Implementation measures	HCP Support package use
Context						
Outer Setting						
Evolving Knowledge of LC	X	X				
NHS System	X	X				
Inner Setting						
In/access to LC NHS care	X	X				
Mixed perceptions of LC NHS care	X	X				
Dynamic NHS contexts & demands			X			
Diversity of LC care	X	X	X			
Participant Factors						
Recovery hopes and expectations	X	X	X			
Ability to research & self-manage	X	X				
Personal environment	X	X				
Heterogeneity of LC	X	X	X			
HCP Factors						
Openness and willingness for change			X			
Knowledge, confidence and preparedness for LC	X	X	X			X
Delivery alongside clinical role			X		X	
Intervention & Implementation						
Flexibility for accessibility and participation		X				
High quality intervention		X			X	
Handbook as a ‘start point’		X				
Fidelity of delivery		X	X	X		X
Support for the realities of delivery			X			X
Mechanisms of Impact						
Prescribed medication/ treatment	X					
Time	X	X				
Continuity of care	X	X				
Being listened to	X	X				
Recognition and validation	X	X				
Safe space to reflect/monitor		X	X			
Personalised approach	X	X	X			
Feelings of progress/ control		X				
HCP connection		X				
Reported Outcomes and Impacts						
Changes in mentality, wellbeing and mood	X	X				
Changes in knowledge and confidence to manage	X	X				
Family/friends’ understanding		X				
Changes in symptom control and day-to-day activity	X	X				
